# Synergistic *in-vitro* activity of Fosinopril alone and in combination with imidocarb dipropionate against *Babesia bovis*

**DOI:** 10.3389/fvets.2026.1899505

**Published:** 2026-08-26

**Authors:** Biswajit Bhowmick, Paul A. Lacy, Carlos E. Suarez, Chungwon J. Chung

**Affiliations:** 1 Department of Veterinary Microbiology and Pathology, Washington State University, Pullman, WA, United States; 2 Animal Disease Research Unit, USDA-ARS, Pullman, WA, United States

**Keywords:** bovine babesiosis, combination therapy, drug repurposing, Fosinopril, fractional inhibitory concentration, imidocarb dipropionate, MTT assay

## Abstract

Bovine babesiosis, caused by *Babesia bovis* is a major cattle disease worldwide, particularly in tropical and subtropical regions. A commonly used drug for the control and treatment of babesiosis is imidocarb dipropionate (ID), which has proved effective against *B. bovis*. Nevertheless, its toxicity to ruminants and other mammals, along with the persistence of drug residues in meat and milk for several months after treatment, has led to restriction on its use. Furthermore, several reports indicate that complete parasite elimination is not obtained in some cases. Interestingly, the angiotensin-converting enzyme (ACE) inhibitor Fosinopril has been recently found to exhibit potent antibabesial activity against *Babesia duncani*. In this study, we investigated the in-vitro activity of Fosinopril alone and in fixed-ratio combinations with ID against the *B. bovis* Texas T2Bo isolate. Quantitative dose–response analyses of the in-vitro activity showed that Fosinopril exhibited measurable activity against the *Babesia bovis* T2Bo isolate, with an IC_50_ value of 541.2 nM, whereas ID demonstrated greater potency, with an IC_50_ value of 124.3 nM. When fixed-ratio combination studies were conducted to evaluate interactions between two drugs, the low-dose regimen Comb-4 (108 nM Fosinopril + 25 nM ID) was the only tested combination that met the predefined ΣFIC criterion for synergy (ΣFIC = 0.40). MDBK cells viability testing showed that ID reduced cell viability at the parasite-relevant concentration tested, whereas Fosinopril and the low-dose combination treatments maintained high MDBK cell viability under the same in-vitro screening conditions. Overall, these proof-of-concept in-vitro findings suggest that Fosinopril has potential as a repurposed therapeutic agent for the treatment of *B. bovis*-infection in cattle. Low-dose Fosinopril–ID combinations may provide a dose-sparing direction for future pharmacological studies.

## Introduction

Bovine babesiosis is a widespread, persistent and serious tick-borne disease that affects cattle and other bovids worldwide. The causative agents of this disease are apicomplexan parasites of the genus Babesia and are primary pathogens for cattle within the genus. Of these species, *Babesia bovis* is considered one of the most pathogenic Babesia species affecting susceptible hosts. In these animals, infection is typically acute and severe, often leading to anemia, neurological dysfunction, circulatory collapse, and death (1–3). Babesiosis occurs through tropical and sub-tropical regions worldwide and, like many parasitic diseases, imposes a substantial burden on livestock production systems in endemic areas due to the cost associated with control measures (3–5).

Imidocarb dipropionate (ID) is one of the most commonly used drugs to treat babesiosis and is often considered as reference drug in in-vitro efficacy studies of Babesia species (3, 6, 7). ID has, however, several adverse effects on treated animals (7–9). Furthermore, residues of imidocarb have been detected in the edible tissues of treated cattle, and its prolonged depletion period may lead to toxicological effects and food safety concerns (10–12). To overcome some of the problems caused by imidocarb, alternative compounds as well as methods to reduce the dose of ID while maintaining its efficacy have been studied (3, 6, 7). In-vitro studies have recently been conducted to identify potential therapeutic candidates against Babesia species and other piroplasmid parasites, as well as to evaluate drug combinations. Buparvaquone and endochin-like quinolones have been shown to be active against *B. bovis*, *B. bigemina,* and other vertebrate hemoparasites. Buparvaquone has been shown to be more effective than imidocarb in several in-vitro evaluations against *B. bovis*. In addition, several drug combinations have been observed to be more effective than single-drug treatments for several endpoints in in-vitro growth assays of *B. bovis* and *B. bigemina* (6, 27). In-vitro assays of parasite growth, together with assessment of post-treatment parasite survival, are therefore valuable tools for identifying potential therapeutic candidates against *B. bovis* infection in cattle and for evaluating their potential efficacy (3, 6, 13–15). Another approach to find new antiparasitic drugs is drug repurposing or repositioning, i.e., the new therapeutic uses of approved drugs. Fosinopril, an FDA approved prodrug for the treatment of hypertension and heart failure as an angiotensin-converting enzyme (ACE) inhibitor (16), has recently been identified as a potent in-vitro inhibitor of *Babesia duncani* (17). The study found that Fosinopril exhibited inhibitory activity against *B. duncani* in the nanomolar range and was more potent than its active metabolite, Fosinopril. Furthermore, Fosinopril activity was found to depend on activation by a parasite-encoded esterase, BdFE1. Given the close relationship between *B. duncani* and *B. bovis*, the species relevant to the present study, Fosinopril may have potential for treating *B. bovis* infection; this plausibility requires experimental verification. The activity of Fosinopril against *B. duncani* and *B. bovis* is biologically plausible beacuse BdFE1 shares homology with predicted esterases in *B. bovis*, *B. microti*, *B. divergens* and *T. equi* (17). However, sequence similarity among organisms does not necessarily imply functional conservation; therefore, further investigation into Fosinopril activation in *B. bovis* cultures is needed (3, 17).

Another area for investigation is the potential combined use of Fosinopril and Imidocarb. Although Fosinopril and imidocarb are two pharmacologically distinct drugs with different modes of action (17, 18), the use of sub-therapeutic levels of imidocarb may reduce its toxicity and the risk of drug residues while enhancing the activity of Fosinopril against *B. bovis* (8, 10–12). However, this would only be beneficial if combining the drugs with subtherapeutic level of imidocarb did not increase toxicity to host cells (6, 7, 19). Therefore, evaluating both the ability of the drug combination to inhibit parasite growth and its potential toxicity to host cells is critical for determining the pharmacological relevance of Fosinopril–imidocarb combinations (7, 19).

In this study we examined the in-vitro activity of Fosinopril, both alone and combination with ID, in cultures infected with Texas T2Bo strain of *B. bovis*. We hypothesized that Fosinopril would exhibit measurable in-vitro activity against *B. bovis* and that selected low-dose combinations of Fosinopril and ID would enhance inhibition of parasite replication while reducing cytotoxicity to host cells. To test this hypothesis, we assessed percent survival in cultures treated with Fosinopril and/or ID, generated dose–response curves, and calculated IC₅₀ values for each treatment. In addition, we determined fractional inhibitory concentrations (FICs) and assessed drug interactions using isobologram analysis for all Fosinopril–imidocarb combinations. Finally, we evaluated cytotoxicity in host cells using an MTT assay on Madin–Darby bovine kidney (MDBK) cells treated with Fosinopril and/or ID. Together, these data provide a basis for evaluating Fosinopril as a repurposed antiparasitic and for identifying promising Fosinopril–Imidocarb combinations for further pharmacological and *in vivo* studies. Because Fosinopril is not approved for clinical use in cattle or other food-producing animals, any translational application would require target-animal pharmacokinetic/pharmacodynamic, safety, residue-depletion, and regulatory evaluation before clinical use could be considered.

## Materials and methods

Drug compounds: Fosinopril sodium (> = 98% purity, HPLC grade) was obtained from Sigma-Aldrich. Imidocarb dipropionate (ID; VETRANAL™, Supelco®, Sigma-Aldrich Chemie GmbH, Buchs, Switzerland) was used as the reference anti-babesial compound. Stock solutions of the various test compounds were made up in the appropriate solvent and then diluted to make working solutions in parasite culture medium on the day of the experiment. Solutions were made immediately before use and added to the parasites.

In-vitro cultivation of *Babesia b*ovis: *B. bovis* Texas T2Bo strain was maintained under long-term microaerophilous stationary-phase culture conditions at 37 °C in an atmosphere of 5% CO_2_, 5% O_2_, and 90% N_2_, as previously described (20). To establish new cultures, *B. bovis* was propagated in 96-well culture plates (96-well culture plate) using 180 μL of PL culture medium per well. The PL medium had a pH of approximately 7.2 and consisted of a 40:60 (v/v) mixture of F-12 K Nutrient Mixture supplemented with L-glutamine and of Iscove’s Modified Dulbecco’s Medium. The medium also included a solution of TAPSO and the antibiotic-antimycotic solution. In addition, the medium contained calcium chloride (anhydrous), antioxidant supplement, insulin, transferrin, and selenium. The complete medium was supplemented with 40% (v/v) of bovine serum and with bovine erythrocytes at a final packed cell volume of 10%.

In-vitro growth inhibition and drug efficacy assay: The study started with a low level of parasitism in the cultures at about 0.8% parasitized red cells (PPE). The drugs, Fosinopril and ID, were tested individually at four concentrations (10 nM, 100 nM, 500 nM and 1,000 nM), as well as in pre-determined combinations, and were added to the cultures for 72 h (the primary phase of the study). The cultures were then monitored for an additional 96 h to assess the extent of parasite clearance following the end of treatment (herein referred to as the post-treatment phase of the study). At the end of the 96 h period, the medium was replaced with fresh medium supplemented with uninfected red cells, and the cultures were allowed to recover and grow for an additional 72 h. This allowed assessment of the level of parasitism following removal of the drug from the cultures. Vehicle-treated infected cultures served as positive controls, while uninfected bovine erythrocytes served as negative controls. Following each medium change, the cultures were incubated for 24 h before parasitemia was determined. A 1 μL aliquot of the resuspended culture was collected to prepare a blood smear and staining using Hema 3 stain. The slides were evaluated for percentage parasitemia (PPE) by counting the number of infected erythrocytes among 5,000 erythrocytes per slide. In addition, parasite morphology was assessed. IC_50_ values for the compounds tested were calculated at 72 h. At this time point, the untreated control cultures were in the exponential growth phase and therefore not at culture plateau. Including culture growth at later time point would have introduced bias into the IC_50_ calculation. Morphological observations were qualitative only and were not used as a primary quantitative endpoint or formal structural scoring system. The in-vitro growth-inhibition design and post-treatment monitoring were based on established *B. bovis* culture methods and recent piroplasm drug-efficacy assays (3, 6, 19, 20). Drug combination and synergy analysis: To examine potential interactions between Fosinopril and ID, fixed-ratio drug combinations were tested doses corresponding to fractions of the respective single agent IC_50_ values measured at 72 h. The dose–response curves were analyzed for both single agents and the IC_50_ values were determined and used for the interaction calculations (Table 1). IC_50_ values of 541.2 nM for Fosinopril and 124.3 nM for ID were determined at 72 h. Fractional Inhibitory Concentration (FIC) were calculated for each drug, and the sum of FIC values (ΣFIC) was subsequently determined. Synergistic interactions are defined as ΣFIC < 0.5, additive or indifferent interactions as 0.5 < ΣFIC ≤ 1, and antagonistic interactions as ΣFIC > 1. This experiment was designed as a focused fixed-ratio interaction screen rather than a complete checkerboard matrix; therefore, FIC values are reported only for the tested concentration ratios. FIC values were calculated as FIC_F = [Fosinopril concentration in the combination]/[single-agent Fosinopril IC_50_]_._

**Table 1:** Combinations of fixed-ratio concentrations of Fosinopril and imidocarb dipropionate used for in-vitro drug-interaction studies to analyze antibabesial activity.

Combination	Fosinopril concentration	Imidocarb dipropionate concentration	Design rationale	FIC value and Interpretation
Comb-1	270 nM	62 nM	Balanced mixture; approximately 1/2 IC_50_ of each drug	FIC_F = 0.50; FIC_ID = 0.50; ΣFIC = 1.00; additive/indifferent
Comb-2	135 nM	62 nM	Imidocarb-dominant mixture; lower fosinopril with 1/2 IC_50_ imidocarb	FIC_F = 0.25; FIC_ID = 0.50; ΣFIC = 0.75; additive/indifferent.
Comb-3	270 nM	31 nM	Fosinopril-dominant mixture; 1/2 IC_50_ fosinopril with lower imidocarb	FIC_F = 0.50; FIC_ID = 0.25; ΣFIC = 0.75; additive/indifferent.
Comb-4	108 nM	25 nM	Low-dose mixture; approximately 1/5 IC_50_ of each drug	FIC_F = 0.20; FIC_ID = 0.20; ΣFIC = 0.40; synergistic.

Fosinopril and imidocarb dipropionate were mixed at fixed-ratio concentrations corresponding to one-half of the IC_50_-values of the respective single compounds determined at 72 h (Comb-1). In addition, imidocarb-dominant (Comb-2) and Fosinopril-dominant (Comb-3) mixtures as well as a low-dose combination (one-fifth of the IC_50_-values of the respective single compounds) (Comb-4) were tested for their potential to increase antibabesial activity while reducing imidocarb exposure. These combinations represent selected fixed ratios and not a full checkerboard concentration matrix. FIC_F, FIC_ID, ΣFIC values, and interaction interpretation are included for each tested combination.

MTT cell viability assay: MDBK cells were used to test the cytotoxicity of Fosinopril, ID and combinations of Fosinopril and ID, in an MTT cell proliferation assay (Abcam, Catalog #ab211091). Cells were added to 96 well plates at a concentration of 500–600 cells per well and allowed to adhere overnight. DMEM medium was then replaced with fresh DMEM with penicillin and streptomycin medium (Cytiva, Logan, UT, USA) containing Fosinopril (540 nM), ID (120 nM), or one of the four test combinations, and the cells were incubated for an additional 72 h. Viability was subsequently assessed using the MTT assay by comparing the absorbance of treated cells with that of untreated control cells. Cell-free medium and vehicle (solvent only) treated cells were used as background and solvent controls, respectively. After the 72 h incubation period, the medium was removed from the wells, and the cells were incubated for an additional 72 h in serum-free medium. Next, 50 μL of the medium was added to each well, followed by 50 μL of MTT reagent. The plates were then incubated at 37 °C for 3 h, after which the medium was removed from the wells and 150 μL of MTT solvent was added to each well. The plates were then incubated in the dark for 15 min with gentle shaking to allow dissolution. The absorbance was read at 590 nm with background subtracted. The results were expressed as a percentage of the viability of untreated cells with cytotoxicity being calculated as 100 – % viability. This assay was used as a preliminary screen to assess cytotoxicity in a bovine cell line.

Statistical Analysis: Analysis was conducted using GraphPad Prism, and all parameters from treated samples were presented as mean ± SD of the three replicate wells. Dose response curves for Fosinopril and ID were generated using non-linear regression, and the IC₅₀ values were determined after 72 h. The IC₅₀ values for single drugs were then used to calculate the fractional inhibitory concentration (FIC) for combination studies. Isobologram graphs were created to assess interactions between Fosinopril and ID. For MTT assays, background absorbance was subtracted from sample readings, which were then normalized to those of untreated control cells to determine % cell viability. Statistical analysis of treated groups was conducted using one-way ANOVA followed by Tukey’s multiple comparisons test. Significant differences were determined at *p* < 0.05. The reported IC_50_ values are point estimates from the tested concentration range; confidence intervals were not calculated because the number of concentration points around the transition region was limited.

## Results

**Figure 1:**
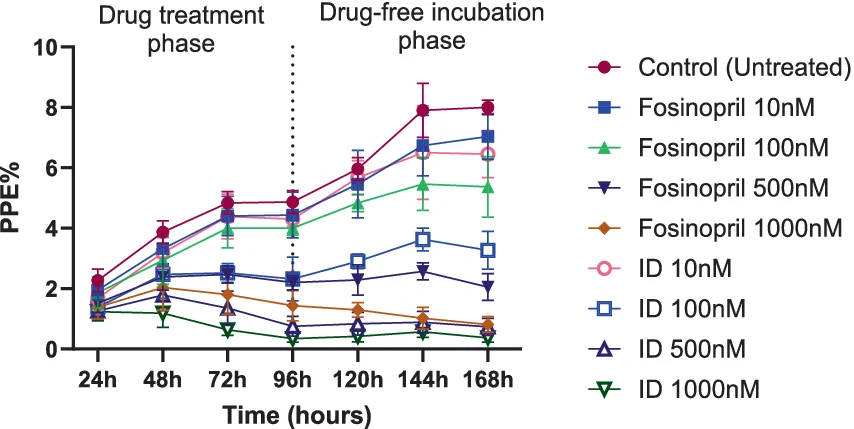
Time-course effect of Fosinopril and imidocarb on *Babesia bovis* parasitemia. Parasitemia (PPE%) was monitored from 24 to 168 h in untreated cultures and cultures treated with Fosinopril or imidocarb at concentrations of 10, 100, 500, and 1,000 nM. The dashed vertical line denotes the transition between the drug treatment phase and the subsequent drug-free incubation phase after culture refreshment. Values are presented as means, with error bars representing the standard deviation of triplicate cultures.

Fosinopril and ID differentially suppressed the growth of *B. bovis* over time. Untreated control cultures showed progressive parasite expansion during the first 72 h of culture, followed by a plateau at 96 h. Following medium replacement and erythrocyte renewal at 96 h, control cultures exhibited an increase in parasite load during the subsequent drug-free observation period. Fosinopril reduced parasite growth in a concentration-dependent manner, with the maximum effect observed at 500–1000 nM (Figure 1). Treatment with Fosinopril at 1000 nM resulted in sustained suppression of parasitemia, even during the subsequent drug-free period. ID produced a more rapid and pronounced reduction in parasitemia during the treatment phase, particularly at 500 and 1,000 nM. However, following treatment with ID, parasites were able to recover during the drug-free phase in several treated cultures, indicating that suppression of parasite growth parameters does not necessarily reflect complete clearance. At low concentrations, both drugs permitted continued parasite growth (Figure 1).

**Figure 2:**
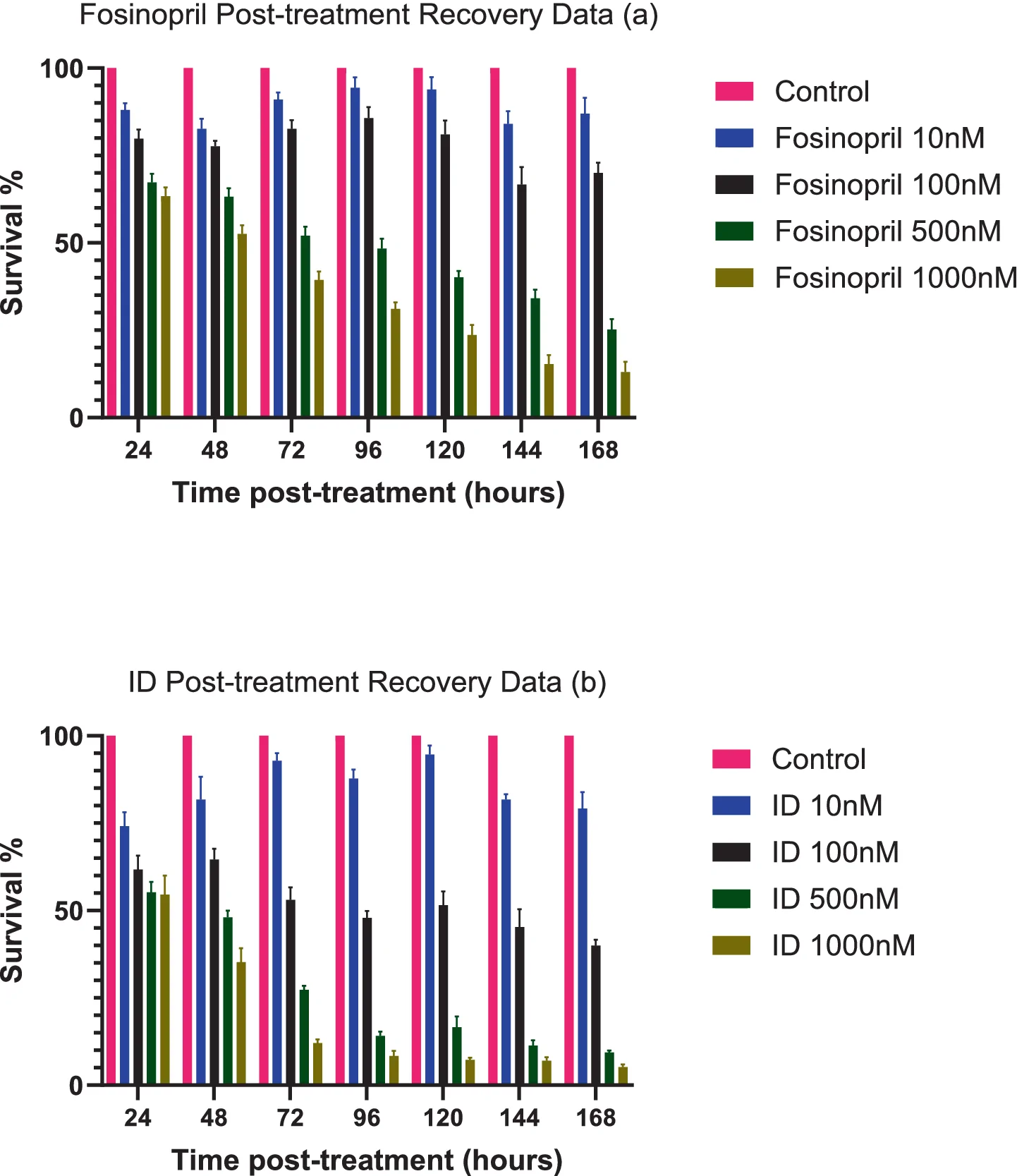
Time-course normalized parasite recovery of *Babesia bovis* after treatment with Fosinopril and imidocarb. Post-treatment recovery (%) is presented relative to control cultures at the corresponding time point. Fosinopril **(a)** and imidocarb **(b)** showed concentration-dependent reductions in parasite survival. Data are presented as means, with error bars representing the standard deviation of triplicate cultures.

Normalized parasite recovery values, expressed as percent post-treatment recovery relative to untreated controls, revealed concentration-dependent inhibition for both drugs. Fosinopril induced a gradual decrease in *B. bovis* survival, with the most pronounced effects observed at later time points at 500 and 1,000 nM (Figure 2a). ID had a more pronounced effect on *B bovis* survival with sharper decreases in parasite survival at all concentrations tested (Figure 2b). Overall, ID exhibited greater potency against the parasite, whereas Fosinopril showed a slower onset of action but sustained inhibition at higher concentrations.

**Figure 3:**
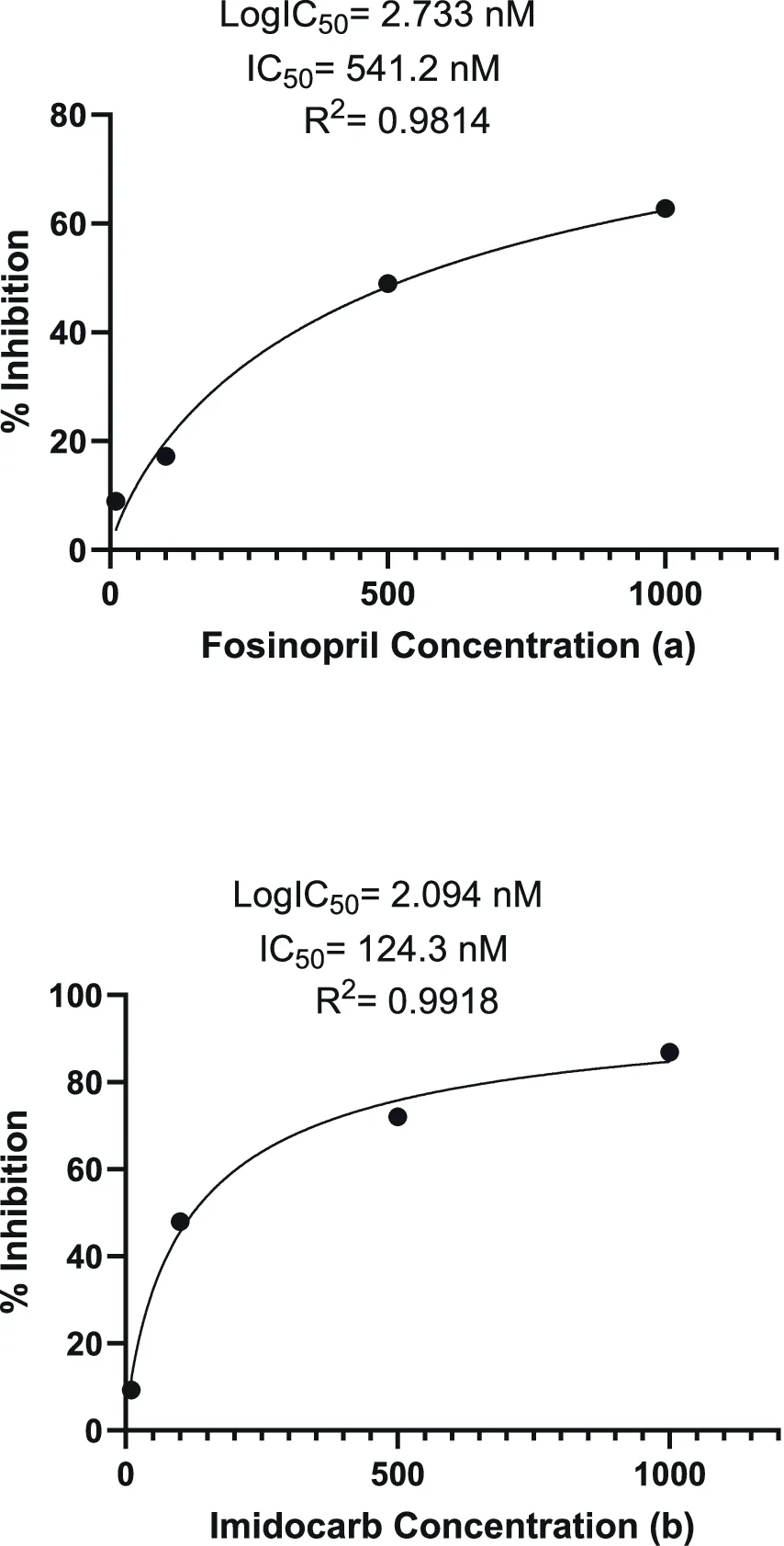
Dose–response curves for Fosinopril and imidocarb against *B. bovis* in-vitro. The percentage inhibition at 72 h of treatment was calculated relative to untreated controls and the curves were fitted by non-linear regression. The calculated IC_50_ for Fosinopril was 541.2 nM **(a)** while for imidocarb it was 124.3 nM **(b)**.

Dose–response analysis revealed a lower IC_50_ for ID compared to Fosinopril: The dose–response study was performed to compare the in-vitro effects of ID and Fosinopril on *B. bovis*. Concentration-response relationships for the in-vitro effects of Fosinopril and ID on *B. bovis* at 72 h post-exposure were analyzed using dose–response methods. For Fosinopril, inhibition of *B. bovis* increased gradually across the tested concentration range, yielding a preliminary estimated IC₅₀ of 541.2 nM (R^2^ = 0.9814; Figure 3a). In contrast, ID showed a steeper dose–response and achieved high levels of inhibition at lower concentrations with an IC_50_ of 124.3 nM (R^2^ = 0.9918, Figure 3b). Thus, ID was more potent than Fosinopril under the tested in-vitro conditions. Because the dose–response analysis was based on a limited number of concentration points, the IC_50_ values should be interpreted as approximate potency estimates rather than highly resolved parameters.

**Figure 4:**
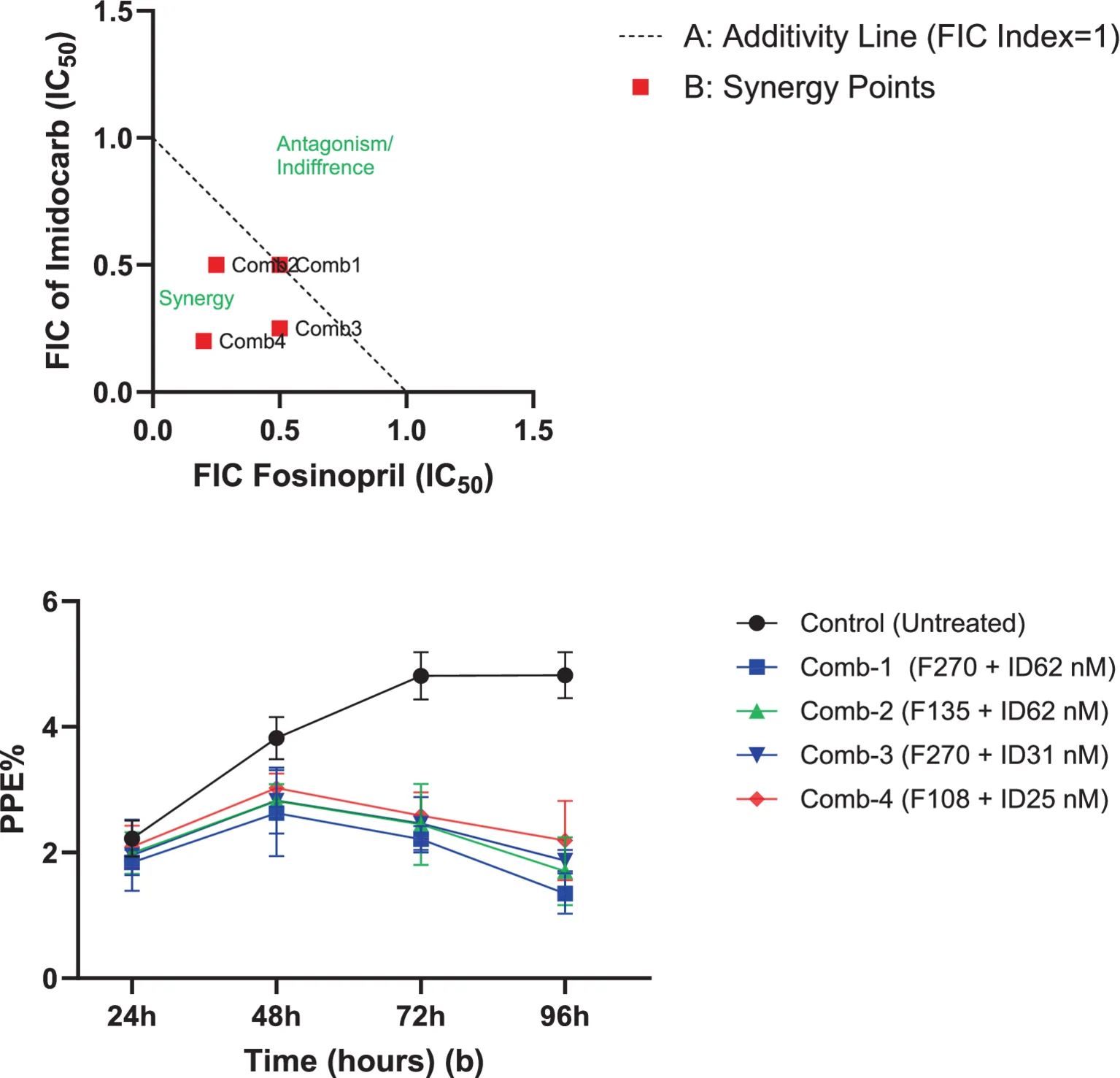
Isobologram of fractional inhibitory concentrations (FICs) for combinations of fixed-ratios of Fosinopril to imidocarb at 72 h post-treatment for *B. bovis* **(a)** and corresponding time-course for percent parasitism inhibition (PPE%) for selected combination treatments **(b)**. The dashed line represents the line of additivity in the isobologram (ΣFIC = 1). Points below the line indicate synergistic interactions. The time-course panel shows PPE% from 24 to 96 h for the same combination regimens compared with untreated controls.

The combination drug cultures were monitored only through 96 h for interaction analysis and were not carried forward into a drug-free recovery phase; therefore, post-treatment recrudescence after combination withdrawal was not assessed. The low-dose Fosinopril-ID combination showed the most favorable interaction among the tested fixed-ratio regimens: Isobologram profiles of the interaction between Fosinopril and ID determined at 72 h after treatment revealed dose-dependent interaction. The isobologram represents selected fixed-ratio points only and should not be interpreted as a complete checkerboard interaction landscape. The drug combination (Comb-1), containing half of the IC_50_ of each drug, was located near the line of additivity, indicating an additive effect. Comb-2 and Comb-3 fell below the line of additivity, indicating moderate synergism. The lowest dose combination of Fosinopril and ID (Comb-4) showed the greatest deviation below the line of additivity and therefore exhibited the strongest synergistic effect (Figure 4a). Time-course analysis of parasite cultures treated with all four combinations showed a reduction in parasitemia relative to untreated controls at each time point from 24 to 96 h (Figure 4b). Although parasite growth occurred during the early phase of treatment, the cultures exhibited less than the untreated controls. Notably, the low-dose combination of Fosinopril and ID achieved the desired balance between reduced drug exposure and sustained inhibition.

**Figure 5:**
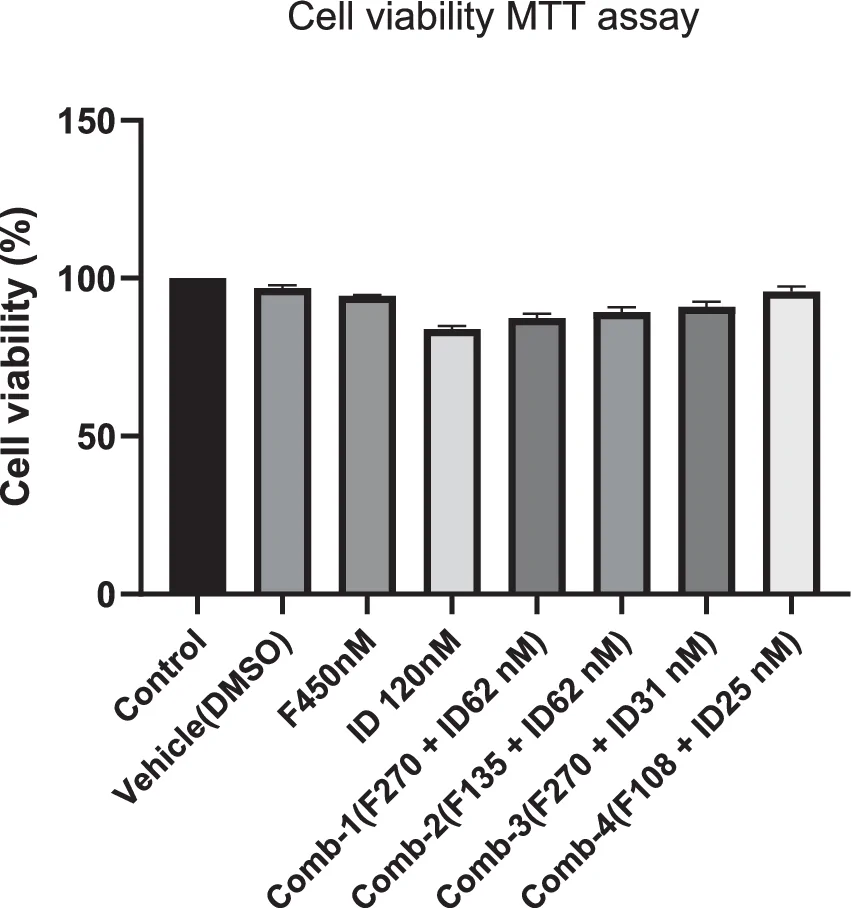
Cytotoxicity of Fosinopril, imidocarb, and their combinations in MDBK cells determined by an MTT assay. MDBK cells were treated with Fosinopril, imidocarb, or combinations of both substances. The viabilities of treated cells were calculated from the background subtracted OD values of the MTT assay in relation to the untreated control cells. Imidocarb reduced cell viability, whereas the low-dose Fosinopril–imidocarb combination (Comb-4) had no effect on viability and was comparable to the vehicle-treated cells.

Low-dose Fosinopril–Imidocarb combination improved the balance between antiparasitic activity and host-cell viability: The MTT analysis of MDBK cells treated with vehicle alone did not result in a decreased viability in comparison to untreated cells (*p* = 0.529), indicating no impact of DMSO in cell viability (Figure 5; Table 2). In addition, Fosinopril alone did not significantly decrease the cell viability of MDBK cells in comparison to vehicle treated cells (*p* = 0.529). However, 120 nM ID significantly decreased MDBK cell viability compared to vehicle (*p* = 0.0002). The in-vitro interactions among the drug combinations exhibited ratio-dependent toxicity. Comb-1 and Comb-2 reduced relative viability to a greater extent than vehicle-treated controls (*p* = 0.0018 and *p* = 0.0062, respectively). Comb-3 produced a smaller reduction in relative viability. Comb-3 produced a smaller but significant reduction in relative viability (*p* = 0.0229). The least effective combination, Comb-4, was not significantly different from the vehicle-treated control group (*p* = 0.9713) and, in fact, exhibited the highest relative viability among all treated groups. Combinations Comb-2, Comb-3 and Comb-4, all significantly increased relative viability compared with ID treatment alone. Comb-4 was the most effective of the combinations tested (*p* = 0.0004). Because a complete dose–response curve for MDBK cytotoxicity was not generated, therapeutic index values could not be calculated. Instead, host-cell viability was evaluated directly at parasite-inhibitory concentrations tested in the same study. Combination cultures were not evaluated during the subsequent drug-free recovery phase; therefore, this does not assess recrudescence following withdrawal of the combination treatment. The isobologram represents selected fixed-ratio points only and should not be interpreted as a complete checkerboard interaction landscape.

**Table 2:** Tukey’s multiple comparisons analysis of MDBK cell viability following treatment with Fosinopril, imidocarb, and Fosinopril–imidocarb combinations.

Tukey’s multiple comparisons test	Mean diff.	95.00% CI of diff.	Summary	Adjusted *p* value
Vehicle (DMSO) vs. F450nM	2.457	−2.644 to 7.558	ns	0.5291
Vehicle (DMSO) vs. ID 120 nM	12.93	7.824 to 18.03	***	0.0002
Vehicle (DMSO) vs. Comb1 (F270 + ID62 nM)	9.385	4.284 to 14.49	**	0.0018
Vehicle (DMSO) vs. Comb2 (F135 + ID62 nM)	7.615	2.514 to 12.72	**	0.0062
Vehicle (DMSO) vs. Comb3 (F270 + ID31 nM)	5.982	0.8810 to 11.08	*	0.0229
Vehicle (DMSO) vs. Comb4 (F108 + ID25 nM)	1.083	−4.017 to 6.184	ns	0.9713
F450nM vs. ID 120 nM	10.47	5.367 to 15.57	***	0.0009
F450nM vs. Comb-1 (F270 + ID62 nM)	6.928	1.827 to 12.03	*	0.0105
F450nM vs. Comb-2 (F135 + ID62 nM)	5.158	0.05687 to 10.26	*	0.0475
F450nM vs. Comb-3 (F270 + ID31 nM)	3.525	−1.576 to 8.625	ns	0.2153
F450nM vs. Comb-4 (F108 + ID25 nM)	−1.374	−6.474 to 3.727	ns	0.9199
ID 120 nM vs. Comb-1 (F270 + ID62 nM)	−3.540	−8.641 to 1.560	ns	0.2122
ID 120 nM vs. Comb-2 (F135 + ID62 nM)	−5.310	−10.41 to −0.2098	*	0.0414
ID 120 nM vs. Comb-3 (F270 + ID31 nM)	−6.943	−12.04 to −1.843	*	0.0104
ID 120 nM vs. Comb-4 (F108 + ID25 nM)	−11.84	−16.94 to −6.741	***	0.0004
Comb1 (F270 + ID62 nM) vs. Comb2 (F135 + ID62 nM)	−1.770	−6.871 to 3.331	ns	0.7996
Comb1 (F270 + ID62 nM) vs. Comb3 (F270 + ID31 nM)	−3.403	−8.504 to 1.698	ns	0.2405
Comb1 (F270 + ID62 nM) vs. Comb4 (F108 + ID25 nM)	−8.301	−13.40 to −3.201	**	0.0037
Comb2 (F135 + ID62 nM) vs. Comb3 (F270 + ID31 nM)	−1.633	−6.734 to 3.468	ns	0.8469
Comb2 (F135 + ID62 nM) vs. Comb4 (F108 + ID25 nM)	−6.531	−11.63 to −1.430	*	0.0145

MDBK cells were treated for 72 h with Fosinopril (F; 540 nM), imidocarb dipropionate (ID; 120 nM) or with several different combinations of Fosinopril and imidocarb. Cell viability was determined by an MTT assay. Means between treatment groups were compared using one-way ANOVA followed by a Tukey’s multiple comparisons test. The results are presented as differences between means, 95% confidence interval, a summary of whether each pair of means were significantly different, and an adjusted *p* value for each comparison. Statistical significance was defined as *p* < 0.05. Ns, not significant; *p < 0.05; **p < 0.01; ***p < 0.001.

## Discussion

The present study aimed to investigate the efficacy of Fosinopril, alone and in combination with imidocarb dipropionate, against cultures of *B. bovis*. The results support the initial in-vitro screening hypothesis in a limited but meaningful way: ID was the more potent single agent, producing more rapid suppression of *B. bovis* growth, whereas Fosinopril was less potent but still producing measurable inhibition of parasitemia. The lowest dose of the Fosinopril–Imidocarb combination tested provided the best balance between antiparasitic interaction and MDBK cell viability.

The inhibitory profile of ID observed in this study supports its status as one of the most widely used drugs for the treatment of bovine babesiosis. Furthermore, ID is considered a reference compound for the in-vitro evaluation of new candidate drugs against this disease (7, 18, 21). ID was more effective than Fosinopril at inhibiting the growth of *B. bovis* in culture. The dose–response curve was steeper and the IC_50_ value was lower for ID than for Fosinopril. These results confirm previous in-vitro studies that demonstrated the efficacy of ID in inhibiting the growth of *B. bovis*. However, the potency of ID in inhibiting the growth of different species of Babesia, as well as different isolates of the same species, may vary (22–24). This variation may be attributed to several factors, including the parasite strain, the initial PPE (%), the culture system used, the concentration range tested, and the assay endpoint (3, 6). In the present study, ID produced a steeper dose–response curve and lower IC_50_ than Fosinopril, indicating higher in-vitro potency against *B. bovis*. Although marked suppression of parasite growth was observed in cultures treated with ID, all cultures revealed signs of parasite regrowth after removal of the drug. Consequently, the present study indicates that marked inhibition of *B. bovis* growth during treatment with ID does not necessarily mean that complete and long-lasting elimination of the parasite is achieved. That *B. bovis* can persist for extended periods in infected cattle and that treatment frequently does not result in complete and lasting elimination of the parasite, the assessment of parasite persistence in culture during post-treatment phase represents an important criterion for evaluating the in-vitro antiparasitic efficacy of drugs against this species (25). Assessing treated parasites can help determine whether the drug kills all parasites immediately or whether some survive for a period of time, potentially leading to persistent infection in cattle. Such persistent infections cannot be eliminated through treatment alone (1, 2, 6, 7, 19). Fosinopril inhibited the growth of *B. bovis* cultures in a dose-dependent manner; however, the inhibitory effect of Fosinopril on growth was less pronounced than that produced by ID. Fosinopril produced more prolonged effect, and cultures frequently harbored low levels of viable parasites at the conclusion of the post-treatment period. This finding is consistent with previous studies that support the use of Fosinopril as a candidate antibabesial agent.

The present study did not directly evaluate fosinoprilat, quantify the conversion of fosinopril to fosinoprilat, or perform knockout, knockdown, or competitive esterase inhibition experiments. Future studies comparing Fosinopril with Fosinoprilat and validating candidate *B. bovis* esterases using biochemical and genetic approaches will be required to determine whether *B. bovis* activates Fosinopril through a pathway analogous to BdFE1 in *B. duncani*. This finding is consistent with previous studies that support the use of Fosinopril as a candidate antibabesial agent. In *B. duncani*, Fosinopril has been shown to inhibit parasite growth at nanomolar concentrations. This activity depends on the conversion of the prodrug to Fosinoprilat by the parasite-encoded esterase BdFE1 (17). Fosinopril activation and esterase activity in *B. bovis* remains to be defined. Moreover, Fosinopril binds strongly to proteins in animals, and using 40% bovine serum in the culture medium for parasites may have affected how much of the drug was actually available in the test. So, the amounts of Fosinopril used in this study cannot be directly compared to the amounts that would be available in the blood of bovine, or how well the drug would work in their systems, or even how well their bodies would absorb it. The interaction seen in this study should be viewed as a sign of synergy, but only for the specific combinations of drugs that were tested. To get a better understanding of how these drugs interact, a more comprehensive study is needed. There are also some limitations to consider. For one, the study only looked at a specific strain of *B. bovis*, and it’s not clear if the results would be the same for other strains found in different parts of the world. Additionally, the study used a type of MDBK cell that is not a perfect model for how the drugs would affect a real animal model. Future studies should use other cell lines that are more similar to those found in a cow’s liver, blood vessels, and other organs to get a better sense of the drugs’ safety. The results of this study should be seen as a preliminary screening for toxicity, rather than a definitive assessment of the drugs’ safety. Furthermore, the concentrations of the drugs used in the study cannot be directly compared to the levels that would be found in a bovine’s bloodstream, because the study did not take into account factors like protein binding and metabolism. Finally, the fact that the combination of drugs was effective at lower concentrations in the study does not necessarily mean that the withdrawal period for meat and milk would be shorter - that would require separate studies to determine. It’s also important to note that the study’s findings are limited to the specific laboratory conditions used. More research is needed to fully understand the potential benefits and risks of using these drugs in combination. The use of Fosinopril and Comb-4 in cattle is complex, and requires careful consideration of multiple factors, including the potential for synergy, the risk of toxicity, and the need for regulated residue-depletion studies. Overall, while the study provides some promising results, it’s just the first step in understanding the potential of these drugs to work together to combat *B. bovis*. Further research is needed to fully explore their potential and to ensure their safe and effective use in cattle. This could involve testing the drugs in different combinations and concentrations, as well as using more advanced models to simulate the real-world conditions under which they would be used. By taking a comprehensive and cautious approach, we can work to develop new and effective treatments for *B. bovis*, while also minimizing the risks to animal and human health. The combination data provide the strongest pharmacological rationale for further development (26). Although the highest drug doses tested achieved the greatest inhibition of parasite growth, they did not necessarily result in the most beneficial drug interactions. Among the doses tested, Comb-4 (the lowest dose) showed the strongest synergistic effect based on isobologram analysis and FIC index calculations. Importantly, combining the drugs enabled effective parasite inhibition at concentrations approximately half those required to reach the IC₅₀ for each drug when used alone. Fosinopril did not induce detectable toxicity in MDBK cells at any of the concentrations evaluated. In contrast, ID was toxic to MDBK cells at all concentrations tested, including the lowest concentration evaluated (100 ng/mL). These results agree with previous studies reporting ID toxicity in veterinary applications (3, 7). These findings support the concept of a dose-sparing strategy that utilize a combination of two drugs for the treatment of *B. bovis* infections and suggest that repurposed antibabesial drug, such as Fosinopril, may have therapeutic potential for managing these infections. The apparent improvement in MDBK cell viability with lower-dose combinations should not be interpreted as a defined host-protective mechanism; rather, it most likely reflects reduced ID exposure under the tested in-vitro conditions. Antiparasitic synergy and reduced host-cell cytotoxicity are therefore related but distinct findings that require separate mechanistic validation. Because Fosinopril is not approved for use in cattle, these data should be viewed as an early in-vitro screening step and not as evidence for near-term clinical translation, altered food-animal withdrawal periods, or regulatory acceptability. The limited number of concentrations used for dose–response fitting also reduces the precision of the IC₅₀ estimates. Future studies should include additional concentrations spanning the transition region and report confidence intervals for the potency estimates.

## Conclusion

The findings in this study support the repurposing of Fosinopril as an antibabesial agent and provide a strong rationale for the use of Fosinopril-Imidocarb combination therapy. A key finding of this study is the identification of a low-dose Fosinopril–ID combination that achieves substantial antiparasitic efficacy while minimizing toxicity to host cells. These results are particularly noteworthy because they suggest, within the limits of the current in-vitro model, that lower ID exposure may be compatible with retained antibabesial activity. These findings are of particular interest and further investigation in both pharmacological studies and *in vivo* experiments. These findings remain preliminary and should be validated using broader drug-interaction matrices, additional *B. bovis* isolates, mechanistic activation studies, and in-vivo pharmacological/toxicological evaluation.

## Data Availability

The original contributions presented in the study are included in the article/supplementary material, further inquiries can be directed to the corresponding author.
